# Effects of metaraminol and norepinephrine on hemodynamics and kidney function in a miniature pig model of septic shock

**DOI:** 10.2478/jtim-2023-0131

**Published:** 2024-07-27

**Authors:** Xiaodan Li, Yi Bai, Ci Tian, Fan Yang, Wenyang Fan, Kuo Zhang, Qingbian Ma

**Affiliations:** Department of Emergency Medicine, Peking University Third Hospital, Beijing 100191, China; Department of Laboratory Animal Science, Peking University Health Science Center, Beijing 100191, China

**Keywords:** septic shock, metaraminol, heart rate, acute kidney injury, conversion dose ratio

## Abstract

**Objective:**

To compare the effects of metaraminol and norepinephrine on hemodynamics and kidney injury in the treatment of septic shock, and calculate the conversion dose ratio between the two vasopressors.

**Methods:**

This randomized controlled study was performed on 15 Guizhou miniature pigs. Septic shock was induced by fecal peritonitis in 10 animals, and 5 were used as a sham-operated group (shams). Fluid resuscitation and vasopressors were initiated 30 min after the onset of septic shock. The septic shock pigs were randomly assigned to receive one of the two drugs to restore and maintain mean arterial pressure (MAP) ≥ 65 mmHg for 3 h. Hemodynamics and heart rate were continuously monitored.

**Results:**

There was no significant difference in MAP, heart rate, cardiac output (CO) and central venous pressure (CVP) between the two groups after treatment. No arrhythmias such as atrial fibrillation and ventricular fibrillation presented during continuous monitoring. After septic shock, the animals showed obvious kidney injury. In addition, compared with norepinephrine, the creatinine at 3 h was significantly lower with metaraminol. According to propensity score matching, the ratio of 6: 1 was considered appropriate for the dose equivalence calculation of metaraminol (μg·kg-1·min-1): norepinephrine (μg·kg-1·min-1).

**Conclusion:**

Metaraminol has a similar pressor effect to norepinephrine in septic shock; it does not increase heart rate and aggravate kidney injury after shock compared with norepinephrine. And our research may provide some laboratory evidence for the clinical usage of metaraminol.

## Introduction

Septic shock is still an important cause of death in critically ill patients, with an estimated incidence of 10.4%^[1]^ and a fatality rate of 40%.^[2,3]^ Vasopressor therapy is an important part of the campaign to save septic shock patients,^[4]^ and norepinephrine (NE) is the first-line vasopressor of choice recommended by sepsis guidelines.^[1,5]^ It increases vascular tone by stimulating α-adrenergic receptors^[1]^ and increases myocardial contractility by stimulating β-adrenergic receptors.^[1,6]^ However, norepinephrine also has potential adverse effects including arrhythmias and visceral circulatory disturbances.^[4,7]^ In addition, norepinephrine has traditionally been administered via central venous catheters (CVCs), mainly due to concerns about peripheral extravasation of vasoconstrictor drugs,^[8]^ thus limiting its emergency use. Metaraminol, which is often used in anesthesia complications, is primarily a selective α1 receptor agonist that has little effect on the heart and can stimulate the release of norepinephrine.^[1,9,10]^ The lack of evidence in international guidelines supporting the use of metaraminol has not limited its use as a first-line agent in clinical practice.^[6,11, 12, 13, 14]^ In large international critical care clinical trials, clinicians used metaraminol as part of concurrent therapy for shock management in 15% to 33% of patients.^[11,15, 16, 17]^ Metaraminol can be administered peripheral intravenously, and studies have shown that there are application scenarios of conversion of metaraminol to norepinephrine in clinical practice.^[11]^ Yet the conversion dose ratio is unknown. More importantly, the kidney is the most commonly affected organ in shock,^[18]^ and vasopressors may aggravate kidney injury. The effects of metaraminol on kidney function, cardiac function, and hemodynamics are not clear. Thus, we used clinically relevant large animal septic shock models to investigate the influence of metaraminol on cardiac function, kidney function, and hemodynamics, and explore the norepinephrine conversion dose ratio.

## Materials and methods

### Ethics statement

All animals (male Guizhou miniature pigs weighing 29–34 kg) were housed in separate cages at 23℃–28℃ and 30%– 60% humidity, with a 12-h light/12-h dark cycle and free access to food and water. All experiments were subjected to approval by the Faculty of Medicine of University of Peking Animal Ethics Committee (LA2019002, 2019/1/14) and performed according to local guidelines.

### Septic animal models

**Figure 1:**
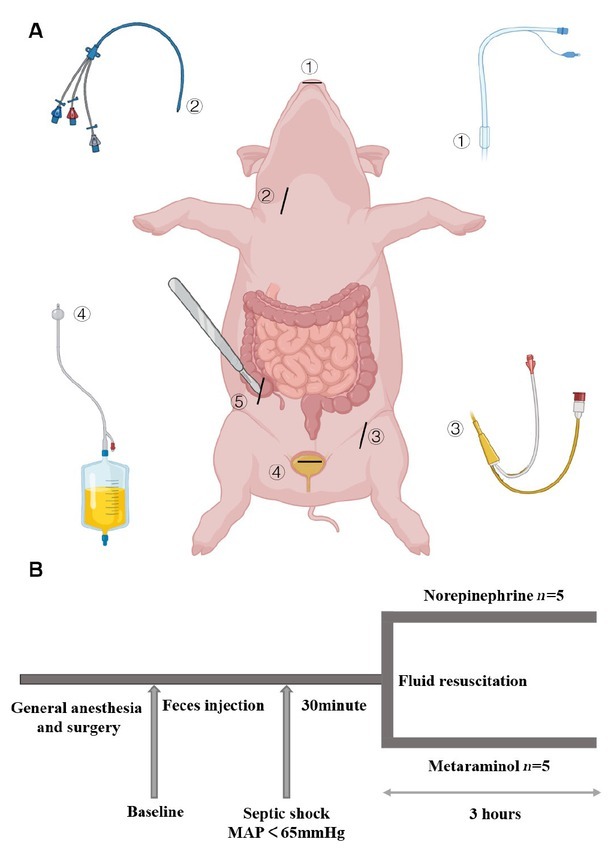
Arrangements for the operation (A) and protocol timeline (B). Endotracheal intubation. Triple lumen central venous catheter. PICCO. Cystostomy. Access to autologous feces from the colon where it joins ④ ⑤ ② ③ ① the cecum. At the end of the operation and after hemodynamic stabilization, baseline measurements were obtained. Blood pressure and heart rate were monitored continuously. The time point at septic shock was defined as the mean arterial pressure (MAP) < 65 mmHg. After 30 min, fluid resuscitation was started with continued infusion of norepinephrine or metaraminol as previously randomized. The last time point was 3 h after the administration of pressor drugs. PICCO: pulse-indicator continuous cardiac output.

This is an open-label, randomized controlled study. Fifteen male Guizhou miniature pigs were used for this study. Experimental protocols were similar to those previously described in similar research,^[19]^ with slight modifications. Briefly, Pulse-indicator Continuous Cardiac Output (PICCO) Monitoring Kits (PULSION Medical Systems SE, Germany) and triple lumen central venous catheters (CVC) (Arrow International, Inc., Czech Republic) were inserted in the femoral artery and internal jugular vein, respectively, to monitor hemodynamic changes and collect blood samples, and cystostomy was used to drain urine (Figure 1A). The animals were randomly assigned to three groups, using online random number generators (Graphpad Random number calculators, https://www.graphpad.com/quickcalcs/randMenu/). In septic animals (*n* = 10), peritonitis was induced by 1 g/kg of autologous feces gotten from the colon where it joins the cecum. Autologous feces dissolved in 200 mL glucose 5% was instilled into the abdominal cavity. In sham-operated animals (*n* = 5), the autologous feces collection was not performed, but received glucose 5% injection. After surgery, the animals were maintained throughout the entire experiment in anesthesia with assisted ventilation, and the temperature of the warming blanket was maintained at 37℃. A combination of compound sodium chloride injection and glucose 5% solutions was administered continuously throughout the experiment at a combined total rate of 5 mL·kg^-1^·h^-1^. All animals were continuously monitored by two experienced researchers with an intensive care medicine background. Eventually the animals were euthanized under deep anesthesia at 3 h after the shock, blood was collected and stored at -80℃, and the kidney samples were harvested and stored in liquid nitrogen until further analysis.

### Hemodynamic monitoring and vasopressor treatment

Mean arterial blood pressure (MAP), heart rate (HR), cardiac stroke volume, and oxygen saturation were monitored continuously. When the MAP of the 10 experimental animals dropped below 65 mmHg and was maintained for 30 min, we considered them to be in septic shock and began to give crystalloids (30 mL/kg) and vasopressors. The animals were randomly assigned to receive one of the two drugs, norepinephrine or metaraminol, to restore and maintain MAP ≥ 65 mmHg. The 5 animals were given norepinephrine at an initial dose of 0.1 (μg·kg^-1^·min^-1^), which was increased by 0.1 unit every 10 min until the mean MAP ≥ 65 mmHg for more than 30 min. The 5 animals in the experimental group were treated with metaraminol at the initial dose of 1 (μg·kg^-1^·min^-1^), which was increased by 1 unit every 10 min. Monitoring was continued thereafter, and if blood pressure decreased subsequently, vasopressors were continued to be upregulated in the same manner until 3 h after shock (Figure 1B).

### Conversion dose ratio and heart rate variability

The conversion dose ratio between metaraminol and norepinephrine refers to a method previously described in another research.^[20]^ In this study, R 4.0.3 statistical software was used for statistical analysis, MatchIt package was used for propensity score matching, Logistic was used to establish the propensity score model, and the neighbor matching method was used for matching. The matching variables were basal mean arterial pressure, shock time, and basal blood pressure combined with shock time, and the matching caliper value was 0.2. The matching ratio between the experimental group and the control group was 1:1. The conversion dose ratio was calculated using the following formula: metaraminol infusion dose (μg·kg^-1^·min^-1^)/ norepinephrine infusion dose (μg·kg^-1^·min^-1^). The HR after administration of the vasoactive drugs was recorded hourly, and the variation of HR was calculated using SPSS 23.0 software.

### Enzyme-linked immunosorbent assay (ELISA)

 Endothelin-1 (ET-1) was measured by ELISA (EK0945-PO, Boster, Wuhan, China).

### Assessment of kidney functions

Concentrations of serum creatinine were measured using a creatinine assay kit (Nanjing Jiancheng Bioengineering Institute, Nanjing, China).

### Assessment of kidney histology

The kidney tissue isolated from the pigs was washed with phosphate-buffered saline (PBS). After fixation with 10% neutral formalin, the kidney tissues embedded in paraffin, and cut into 4-μm-thick sections for haematoxylin and eosin (HE) staining. HE staining of kidney tissues was examined by microscopy, and the images were analyzed by computerised digital image analysis (NDP view 2.0). The tubular injury was examined by the percentage of damaged tubules ^[21]^: grade 0, no damage; grade 1, < 25%; grade 2, 25%–49%; grade 3, 50%–75%; and grade 4, > 75%.

### Western blotting

The protein was extracted from the kidney tissue with radioimmunoprecipitation (RIPA) lysis buffer containing protease inhibitor in order to protect the protein from degradation. The supernatant was then collected and the protein concentration was measured by a bicinchoninic acid (BCA) protein assay kit. The samples were subjected to SDS-PAGE, and then protein was transferred to blotting membrane. The membrane blocking was then done with 5% nonfat milk and incubated at room temperature for 2 h. The blot was incubated with a specific primary antibody at 4°C overnight, which was followed by incubating the membranes with horseradish-peroxidase (HRP) conjugated secondary antibody for 2 h at ambient temperature. Finally, the membranes were photographed to X-ray film by an enhanced electrochemiluminescence (ECL) substrate kit from Yeasen Biotechnology, Shanghai, China. The relative expression ratios for the control and experimental groups were analyzed based on density by Image J software and the β-Actin signal as a reference. Antibodies were used against KIM-1 (bs-2713R; Bioss, Beijing, China), and β-actin (3700S; CST, Shanghai, China).

### qRT PCR assay

RNA extraction and quantitative real-time (qRT) polymerase chain reaction (PCR) total RNA was extracted using TRIzol Reagent (Ambion, Carlsbad, CA), according to the manufacturer’s instructions, and RNA concentration was determined by Nanodrop. RNA was reverse transcribed in Biometra TAdvanced 96 SG (Analytikjena, Germany). The levels of neutrophil gelatinase-associated lipocalin (NGAL), IL-6, TNF-α, IL 33, CXCL10, COL1A1, and GAPDH were determined by Archimed v1.3.3. The primers are described in this paper’s supplemental material (see Table S1). The relative levels of target genes were analyzed by the 2–ΔΔCt method, normalized to the GAPDH, and compared with controls.

### Statistical analysis

After testing for normality of distribution by the Shapiro-Wilk test, the continuous data were presented as means ± standard deviation (SD) or medians (25th–75th) depending on the distribution. Independent samples Student’s *t*-test and Mann-Whitney U test were used to calculate P values for continuous variables. MAP, heart rate, cardiac output (CO), central venous pressure (CVP), urine output, and creatinine in norepinephrine and metaraminal were analyzed using two-way repeated measures analysis of variance (ANOVA). If the normal distribution and homogeneous variance are not satisfied, the Scheirer-Ray-Hare test was used as an alternative method by using R 4.2.2 with the “Rcompanion” package. The analysis was performed using SPSS version 23.0 for windows (IBM Crop., Chicago, USA). A value of *P* < 0.05 (corrected, 2-sided) was considered to be statistically significant.

## Results

### Septic shock induction

**Figure 2:**
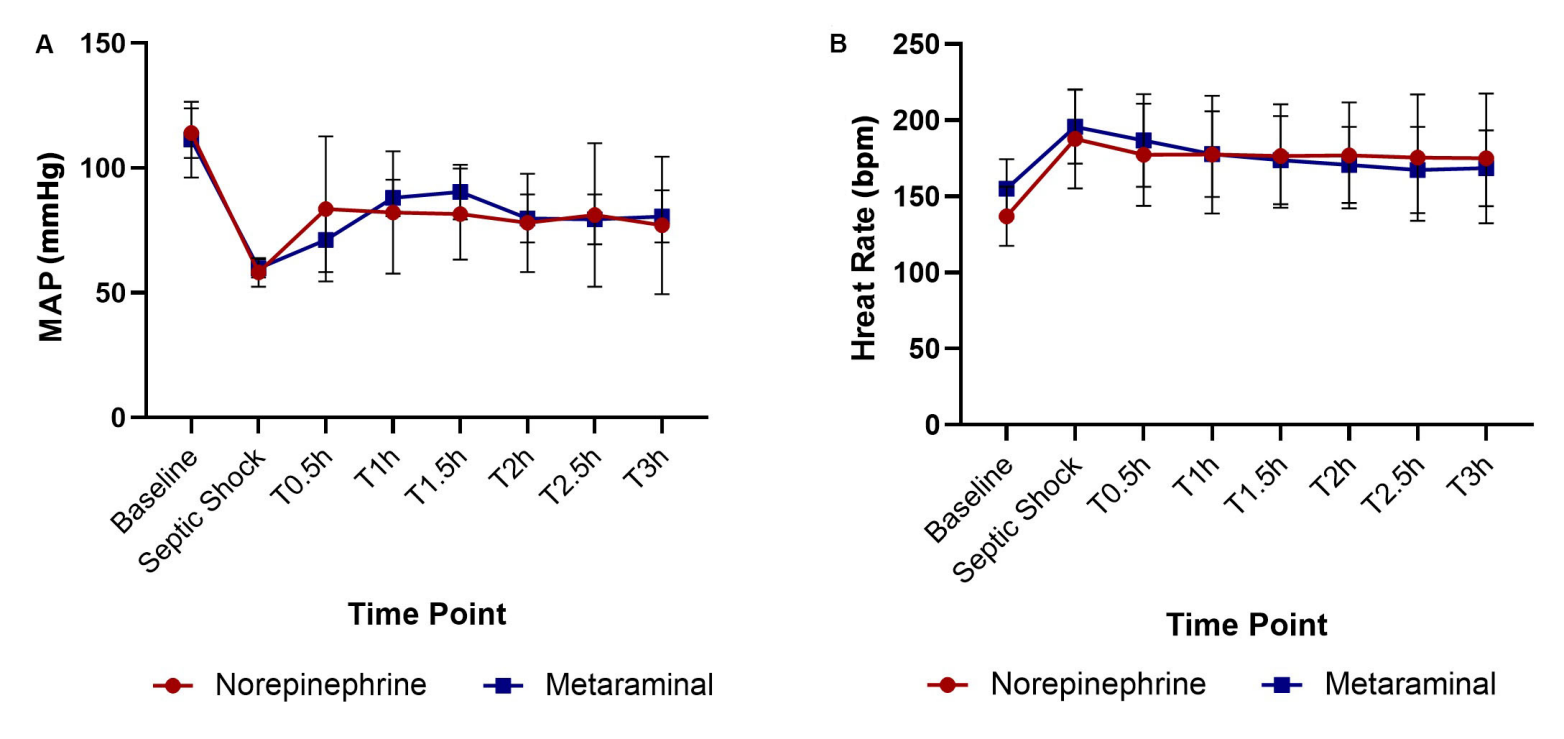
Hemodynamic variables in the three groups. Changes in MAP (A) and heart rate (B) throughout the experiment. T0.5 is 0.5 h after administration of the drugs, T1 is 1 h, T1.5 is 1.5 h, T2 is 2 h, T2.5 is 2.5 h, and T3 is 3 h. MAP: mean arterial pressure.

All 10 animals in the two treatment groups developed severe hypotension (*P* < 0.01) and tachycardia (*P* = 0.006) compared to the baseline (Figure 2A and 2B). The mean time to reach the septic shock timepoint was similar in both treatment groups (*P* = 0.22) (Table 1). There were no statistically significant differences in MAP, HR, CO, global ejection fraction (GEF), or systemic vascular resistance (SVR) between the treatment groups until the administration of the vasopressor (Table 1).

**Table 1:** Hemodynamic variables in the three groups at the baseline (mean ± SD)

Variables baseline		Sham (*n* = 5)	Norepinephrine (*n* = 5)	Metaraminol (*n* = 5)
MAP (mmHg)		122 ± 17	114 ± 10	111 ± 15
HR (/min)		136 ± 7	137 ± 19	1 55 ± 19
T (℃)		33.9 ± 0.7	35.4 ± 1.7	36.0 ± 1.1*
CO (l/min)		2.98 ± 0.75	2.53 ± 0.92	2.53 ± 0.80
GEF (%)		24.2 ± 6.6	16.8 ± 4.3	17.6 ± 3.3
SVR (dyn. s.cm^-5^)		3870 ± 2014.3	4552 ± 1808.7	3926 ± 1087.6
Septic shock time point	(h)	-	15 ± 4.6	21 ± 9.4

MAP: mean arterial pressure; HR: heart rate; T: temperature; CO: cardiac output; GEF: global ejection fraction; SVR: systemic vascular resistance. *P < 0.01 between sham and metaraminol.

### Hemodynamics indices

**Figure 3:**
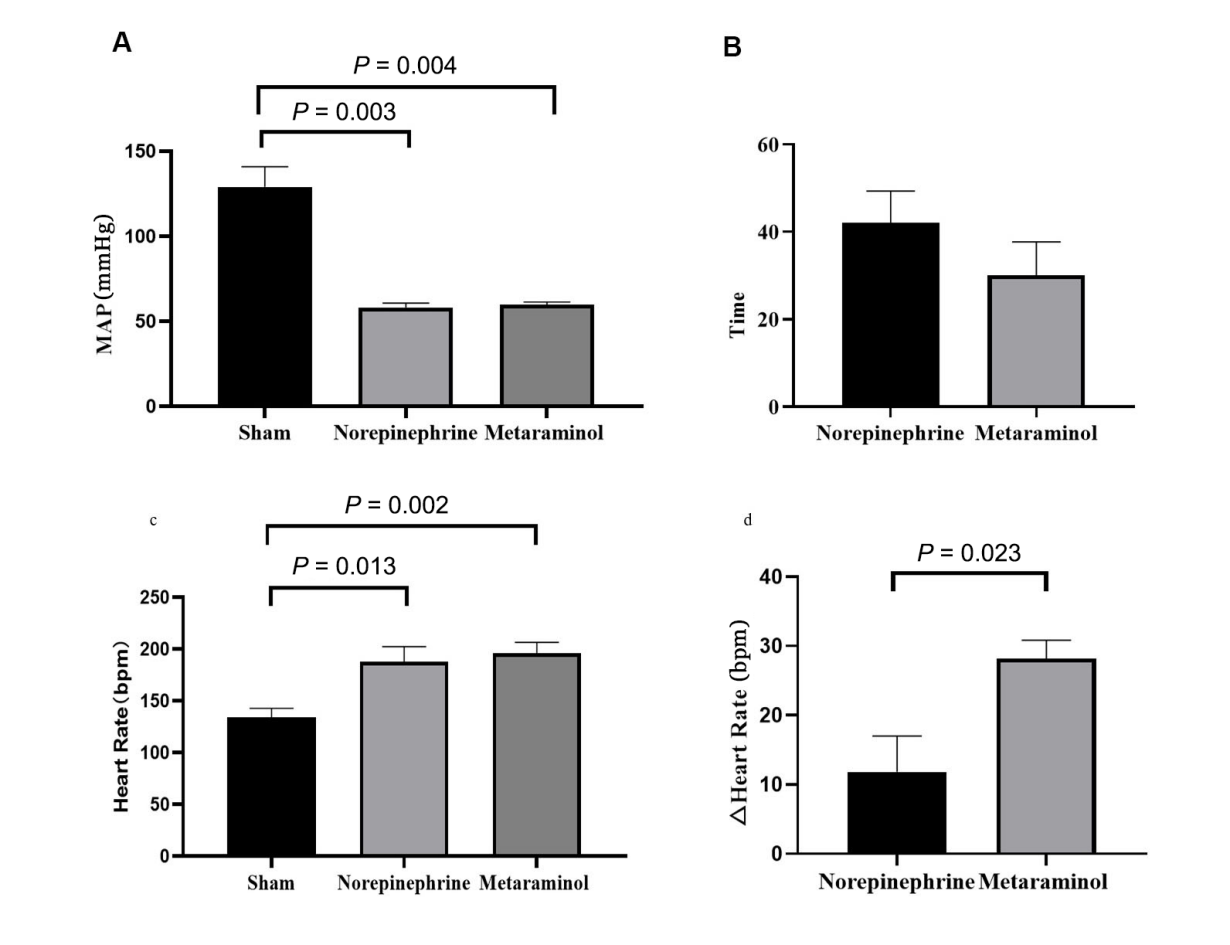
MAP and heart rate in the three groups. A. Comparison of map while in shock in two treatment groups with shams. B. Time to reach MAP ≥ 65 mmHg after shock. C. Comparison of heart rate in shock. D. Changes in heart rate from shock to 3 h after treatment. MAP: mean arterial pressure.

At shock, the MAP was 60 ± 4 mmHg in the metaraminol group, and 58 ± 6 mmHg in the norepinephrine group, which declined significantly than the sham group (Figure 3A). The MAP was restored to 65 mmHg in 30 min with metaraminol and 42 min with NE (Figure 3B), namely the target (MAP ≥ 65 mmHg) blood pressure was reached faster with metaraminol. However, there were no statistically significant differences between the two sepsis groups at every timepoint (*F* = 1.009, *P* = 0.435) (Figure 2A). The mean dose required to maintain MAP ≥ 65 mmHg with NE was 0.38 ± 0.13 μg·kg^-1^·min^-1^, and it was 3.00 ± 1.73 μg·kg^-1^·min^-1^ with metaraminol. The total dose between the septic shock timepoint and the end of the experiment was 70.6 ± 25.5 μg/kg with NE and 510.0 ± 253.4 μg/kg with metaraminol.

The mean HR at shock was 196 ± 24 beats/min in the metaraminol group and 188±32 beats/min in the NE group, which increased significantly than the sham group (Figure 3C). The mean HR at 3 h after shock was 168 ± 26 beats/min with metaraminol and 176 ± 43 beats/min with NE; there were no statistically significant differences between the two treatment groups at every timepoint (*F* = 0.007, *P* = 0.937) (Figure 2B). However, after 3 h of treatment, the HR declined significantly with metaraminol (*P* = 0.023) (Figure 3D). No arrhythmias such as atrial fibrillation and ventricular fibrillation presented during continuous monitoring.

**Figure 4:**
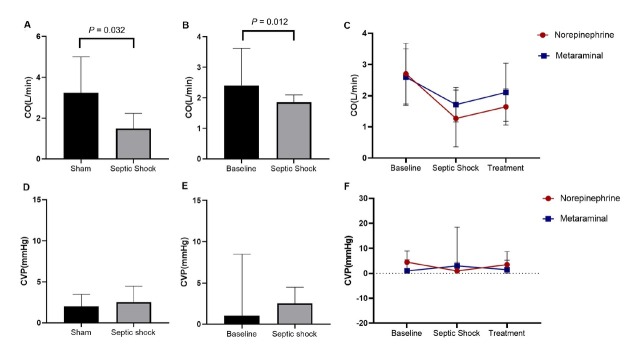
CO and CVP in the three groups. A. Comparison of CO between in sepsis animals and shams. B. Comparison of CO between shock and baseline. C. The dynamic change over time of CO. D. Comparison of CVP in sepsis animals and shams. E. Comparison of CVP between shock and baseline. F. The dynamic change over time of CVP. CO, Cardiac Output; CVP, Central Venous Pressure.

At shock, the CO was 1.71 ± 0.55 L/min in the metaraminol group, and 1.27 ± 0.91 L/min in the norepinephrine group, which declined significantly than the sham group (*P* = 0.032) (Figure 4A) and the timepoint of baseline (*P* = 0.012) (Figure 4B). After treatment, CO was higher in both sepsis groups than in shock. However, there were no statistically significant differences between the two sepsis groups at every timepoint (*F* = 0.293, *P* = 0.608) (Figure 4C). For the CVP, there were no statistically significant differences between the sepsis and sham groups (*P* = 0.794) (Figure 4D), and the time of baseline and shock (*P* = 0.915) (Figure 4E). There were also no statistically significant differences between the two sepsis groups at every timepoint (H = 0.220, *P* = 0.639) (Figure 4F). The change of CVP has no time dependent effect (H = 0.094, *P* = 0.954).

### Evaluation of ET-1 expression

The serum ET-1 concentration for the experimental animals in shock was significantly higher than that of the baseline (*P* = 0.04) (see Figure S1A). There were no statistically significant differences in serum ET-1 concentration between the metaraminol and norepinephrine groups at shock (*P* = 0.261) (see Figure S1B), and nor at 3 h after treatment (*P* = 0.101) (see Figure S1C).

### Evaluation of kidney injury

**Figure 5:**
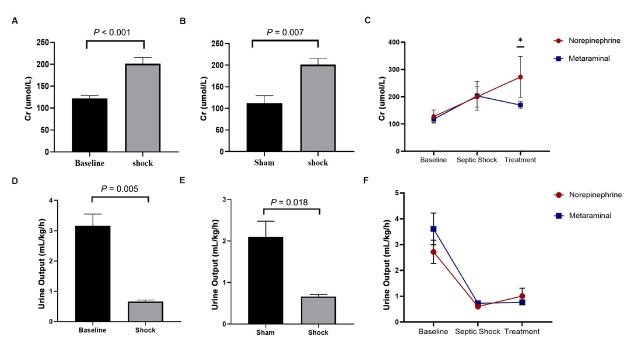
Expression of serum Cr and by shock and treatment. A. Comparison of Cr between shock and baseline. B. Comparison of Cr between in sepsis animals and shams. C. The dynamic change over time of creatinine. D. Comparison of the values of urine output between shock and baseline. E. Comparison of the values of urine output in sepsis animals and shams. F. The dynamic change over time of urine output. Cr, creatinine.

The serum creatinine (Cr) at shock was significantly higher than baseline (*P* < 0.001) (Figure 5A), and was also significantly higher in the sepsis animals than in the shams (*P* = 0.007) (Figure 5B). There were no statistically significant differences between the metaraminol and norepinephrine group in the serum Cr at shock (*P* = 0.909) (Figure 5C). The Cr has different trends over time in the two groups (*F* = 9.890, *P* = 0.014), and the serum Cr in the metaraminol group was significantly lower than in the norepinephrine group after 3 h of treatment (*F* = 34.262, *P* = 0.017) (Figure 5C).

Urine output is very crucial for evaluating renal function. The values of urine output decreased significantly over time (*F* = 30.756, *P* < 0.001). The values of urine output at shock were significantly reduced than baseline (*P* = 0.005) (Figure 5D) and sham group (*P* = 0.018) (Figure 5E), but there were no statistically significant differences between the metaraminol and norepinephrine group in the values of urine output at shock (*P* = 0.251) and treatment (*P* = 0.463) (Figure 5F).

**Figure 6:**
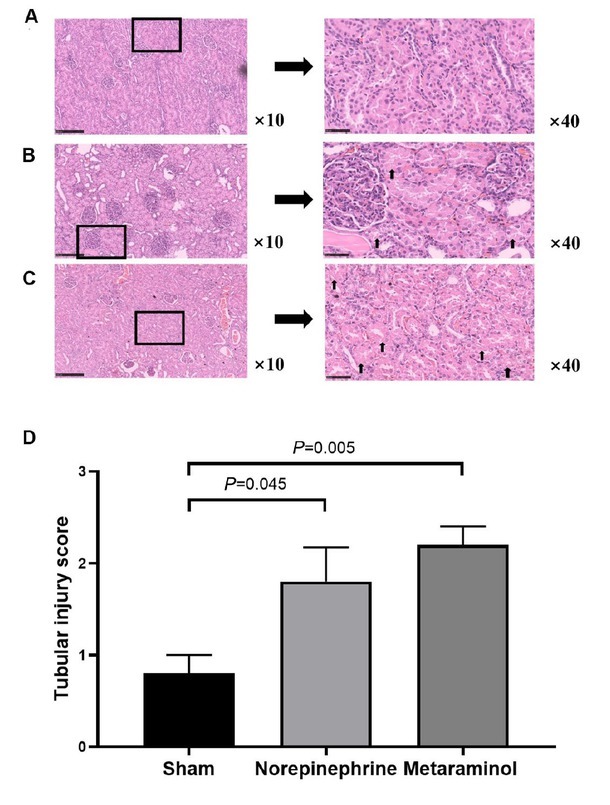
Cardinal features of acute kidney injury. Representative histology and pathological scores of tubular injury in kidney cortex by HE staining in sham group (A), norepinephrine group (B) and metaraminol group (C). The arrows indicate magnified views of the tubular injury. D. The tubular injury score in three groups.

HE staining of the cortex showed that kidney injury with intraepithelial vacuolar degeneration and nucleolus had disappeared and that the inflammatory cells were mainly distributed in the renal sinuses rather than in the renal parenchyma at shock (Figure 6A-6C). Quantitative analysis of the tubular injury score of metaraminol and norepinephrine group renal tissues was 2.2 ± 0.45 and 1.8 ± 0.84, compared with 0.8 ± 0.45 for sham group kidneys. The tubular injury score was significantly higher in the sepsis animals than in the shams (*P* = 0.045 and *P* = 0.005), but there were no statistically significant differences between the metaraminol and norepinephrine group (*P* = 0.343) (Figure 6D).

**Figure 7:**
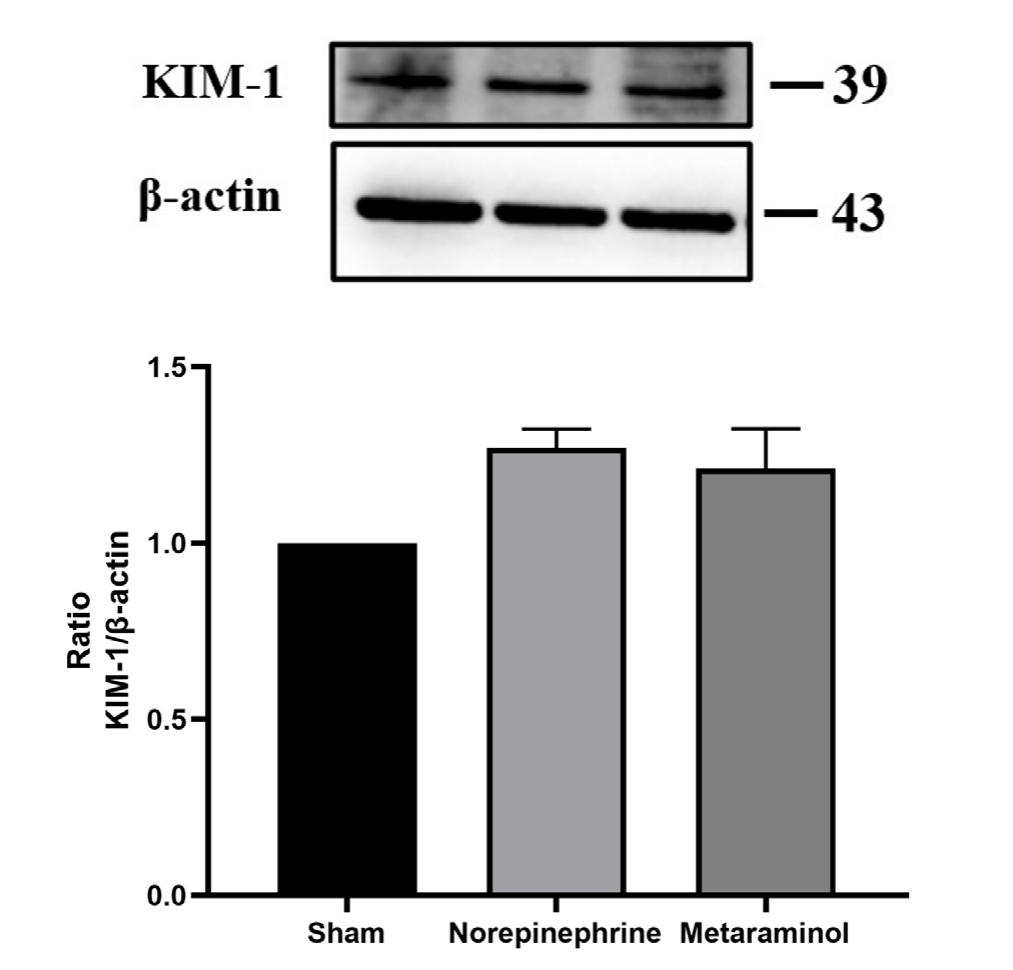
KIM-1 expression in kidney.

**Figure 8:**
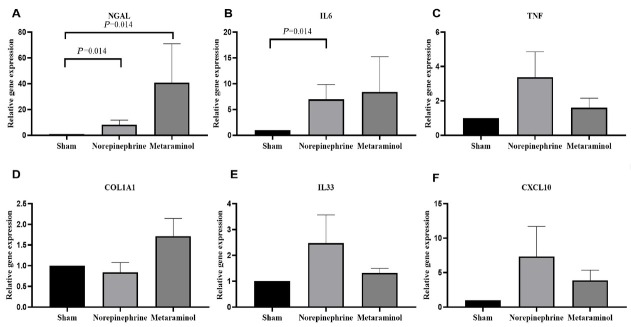
Renal tissue relative mRNA expression of NGAL (A), IL6 (B), TNF (C), COL1A1 (D), IL33 (E), and CXCL10 (F). Relative quantification was achieved using the comparative 2-ΔΔCt method by normalization with the GAPDH. Results are expressed as relative fold increase above the mean value of renal tissue mRNA expression of the sham group, arbitrarily fixed at 1. NGAL: neutrophil gelatinase-associated lipocalin; IL6: interleukin-6; TNF: tumor necrosis factor; COLIA1: collagen Iα1; IL33: interleukin-33; CXCL: chemokine (C-X-C motif) ligand.

The expression of kidney injury molecule 1 (KIM-1) protein, a marker of renal tubular injury, was detected by western blot. The results showed that the expression of KIM-1 protein in the kidney tissue of the sepsis animals was increased compared with that of the shams (*P* = 0.025), but there was no significant difference in the expression of KIM-1 protein between metaraminol and norepinephrine groups (*P* = 0.665) (Figure 7). Shock significantly increased the mRNA expression of NGAL in pig kidney tissue, suggesting the occurrence of acute kidney injury, but there was no statistically significant difference between the two treatment groups (*P* = 0.564). Similarly, mRNA expressions of COL1A1, TNF, IL6, IL33, and CXCL10 in kidney tissues showed no statistically significant differences between the treatment groups (*P* > 0.05) (Figure 8A-8F).

### Conversion dose ratio

The median infusion speed to reach MAP ≥ 65 mmHg was 2 μg·kg^-1^·min^-1^ for metaraminol treatment and 0.4 μg·kg^-1^·min^-1^ for norepinephrine. Based on the basal MAP, the result of the propensity scores of the experimental group and the control group after matching were 5 and 5. The median conversion dose ratio between metaraminol and norepinephrine was 6 (IQR 4–20). Based on the time to reach shock, the scores of the matching were 4 and 4, and the median conversion dose ratio of 7 (IQR 4–30). Based on both indicators, the propensity scores of the matching were 3 and 3, and the median conversion dose ratio was 6 (IQR 4–6.67). Thus, the median ratio is estimated to be between 6 and 7. Pragmatically, this can be estimated to a ratio of 6: 1 for ease of calculations and can be considered appropriate for the dose equivalence calculation of metaraminol (μg·kg^-1^·min^-1^): norepinephrine (μg·kg^-1^·min^-1^).

## Discussion

In this study, metaraminol and norepinephrine combined with optimal fluid administration were both able to maintain blood pressure at normal levels during the first 3 h of septic shock, and the two drugs had similar pressor effects. Through continuous monitoring of heart rate, we found that shock caused a significant increase in heart rate in the animals, which was alleviated observably by the use of metaraminol. There were no significant differences in CO and CVP between the two drugs. The occurrence of acute kidney injury after shock was observed in the increase of serum creatinine and NGAL, and the decrease of urine output, which are markers of kidney injury. Metaraminol significantly alleviated the increase of creatinine. Using the statistical methods of propensity score matching, we determined that a ratio of 6:1 (metaraminol: norepinephrine) can be considered appropriate for use in dose equivalence calculations.

Metaraminol is predominantly an α1 agonist, which is often used to prevent hypotension caused by spinal anesthesia, and is also widely used in the treatment of shock. Metaraminol is not currently recommended in international guidelines for management of septic shock due to the limited research currently available to demonstrate its safety and efficacy.^[22]^ However, studies have found that metaraminol is often used as a first-line peripheral pressor in critically ill patients, and as a single agent in patients with non-refractory shock.^[11,22,23]^ We therefore compared the effects of raising blood pressure with metaraminol and the first-line drug norepinephrine, and found that the two drugs maintained the blood pressure at similar levels, and the effects on CO and CVP showed no difference, a result which was similar to that of previous studies.^[1,24]^ In addition, by monitoring changes in blood pressure in real time and titrating doses of the pressors, we found that the use of metaraminol seemed to raise blood pressure faster than norepinephrine. This phenomenon needs to be further verified with a larger sample size. Our results showed that as blood pressure increased, the increase in heart rate was suppressed significantly with metaraminol. No arrhythmias such as atrial fibrillation and ventricular fibrillation presented during continuous monitoring between those two sepsis groups. Previous studies have found that metaraminol increases blood pressure by stimulating peripheral vascular α receptors, reflexively stimulates vagal tone and prolongates atrioventricular conduction, and terminates tachycardia, and metaraminol has no excitatory effect on myocardium, and it can reduce heart rate and myocardial oxygen consumption by reflexively stimulating the vagus nerve, which has a cardioprotective effect.^[25,26]^ International guidelines for septic shock, in settings where norepinephrine is not available, recommend that dopamine can be used as an alternative.^[4]^ However, dopamine use is limited due to the side effect of increased heart rate and the risk for arrhythmia.^[4,27,28]^ Therefore, metaraminol may be more suitable as an alternative to norepinephrine or as a monotherapy for septic shock.

Acute kidney injury occurs in 60% of sepsis patients,^[18]^ and the use of vasopressors may result in vasoconstriction and increased kidney injury.^[1,29]^ In our study, we selected a panel of markers to capture cardinal features of acute kidney injury, but there were no significant differences in the urine output, tubular injury score, KIM-1, NGAL, cytokine, and COL1A1 between the metaraminol and norepinephrine groups. In addition, the creatinine value at 3 h after treatment was significantly reduced with metaraminol. Thus, metaraminol would not aggravate kidney injury after shock compared with norepinephrine, a result that is similar to the results of previous studies.^[30]^ Prior studies have found that cardiopulmonary bypass leads to renal hypoperfusion, resulting in medullary hypoxia and acute kidney injury, but that if medullary perfusion and tissue oxygen tension (PO^2^) was maintained with low-dose metaraminol, the drug increased perfusion pressure without affecting renal vascular resistance.^[31]^ The mechanism of the potential protective effect of metaraminol on the kidney needs to be investigated further.

Calculation of the equivalent dose ratio between pressors is necessary in clinical practice and in research related to shock management. Previous clinical trials have not included metaraminol in the quantification of total pressor exposure due to lack of evidence for conversion dose ratios.^[17,20,32]^ Using the method of calculation the conversion dose ratio referred to in previous research.^[20,24]^ We found that a 6:1 ratio was appropriate for dose equivalence calculations. One of the studies compared the hemodynamic effects of continuous infusion of metaraminol and norepinephrine in 10 patients with septic shock. The patients were initially given titrated norepinephrine and stopped when their MAP reached 65 mmHg, and then the patients were immediately transitioned to metaraminol. By comparing the infusion doses required to achieve the same blood pressure target, the conversion dose ratio was calculated as 8.3:1.^[24]^ Another clinical study used similar approach, but initially used metaraminol and then transitioned to norepinephrine, resulting in a ratio of 10:1.^[20]^ Patients in both studies received the two drugs in sequence, so the cumulative effect of the initial drug on the second cannot be ruled out. Patients were also treated with other vasoactive drugs, such as cardiac drugs, which may have interfered with the results. In our study, a single agent was used in animals without interference from other agents. Since it was not the same animal, we matched the two treatment groups of five animals in order to calculate the ratio. We used both the basal blood pressure and the time to reach shock after the operation, which represented the basal characteristic of the animals and the impact of the operation, respectively, to calculate the propensity score for matching, and the ratio results were similar. Due to the small sample size, there may be bias, so a larger sample size is needed for further verification. This result, however, provides guidance for clinicians to switch between the use of metaraminol and norepinephrine to avoid hypotension or other complications.

In conclusion, in a resuscitated large animal model of septic shock, metaraminol administration can restore MAP as efficiently as can NE, and it does not increase heart rate and aggravate kidney injury after shock.

## Supplementary Information

Table S1. Primers.

Figure S1. Expression of ET-1 by shock and treatment.

Supplementary information is only available at the official site of the journal at: www.intern-med.com.

## Supplementary Material

Supplementary Material
