# Supplementary material for: Effects of metaraminol and norepinephrine on hemodynamics and kidney function in a miniature pig model of septic shock
Source: J Transl Int Med. 2024 Jul 27;12(3):253–62. doi: 10.2478/jtim-2023-0131 (PMC11284895; doi:10.2478/jtim-2023-0131)
Supplement: Supplementary file 1 — Supplementary Material [file jtim-2023-0131_sm.pdf]

Supplemental Digital Content

Table 1: primers.

Gene		Sequence (5’-3’)
TNF-α	Forward	CCCTCACGTCCTTCTGGTTT
	Reverse	GAGTCTGGAAGCCCCAGTTC
COL1A1	Forward	GTCTGGTTTGGAGAGAGCAT
	Reverse	CTTCTTGAGGTTGCCAGTC
IL-6	Forward	ACAAAGCCACCACCCCTAAC
	Reverse	CGTGGACGGCATCAATCTCA
IL-33	Forward	CTTCATGAGCAGCCCTCCAA
	Reverse	TCCGCAGCTTTCTGTCACAT
CXCL10	Forward	ATAAGGATGGGCCGGAGAGA
	Reverse	GTGGGAGCAGCTAACTTGGT
NGAL	Forward	TTAAGAAATACTCTGGATTGC
	Reverse	TACTCTTGGTTGTTGGAAAC
GAPDH	Forward	TCGGAGTGAACGGATTTGGC
	Reverse	TGACAAGCTTCCCGTTCTCC

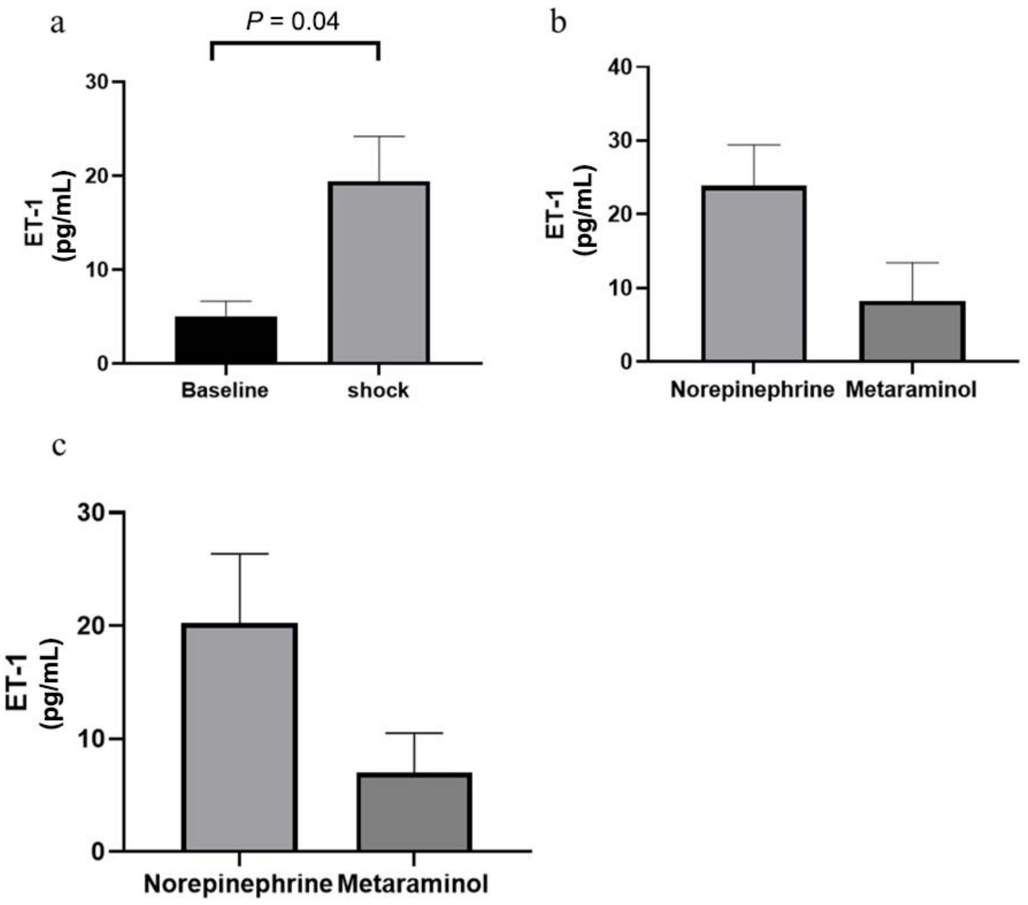

Figure 1: Expression of ET-1 by shock and treatment. 1a: Comparison of ET-1 expression between shock and baseline. 1b: Comparison of ET-1 expression between the two treatment groups in shock. 1c: Comparison of ET-1 expression between the two groups 3 hours after treatment.
